# Complete Genome Sequence of *Paenibacillus polymyxa* CR1, a Plant Growth-Promoting Bacterium Isolated from the Corn Rhizosphere Exhibiting Potential for Biocontrol, Biomass Degradation, and Biofuel Production

**DOI:** 10.1128/genomeA.01218-13

**Published:** 2014-01-23

**Authors:** Alexander W. Eastman, Brian Weselowski, Naeem Nathoo, Ze-Chun Yuan

**Affiliations:** a Southern Crop Protection and Food Research Centre, Agriculture and Agri-Food Canada, London, Ontario, Canada; b Department of Microbiology and Immunology, University of Western Ontario, London, Ontario, Canada; c Department of Biology, University of Western Ontario, London, Ontario, Canada

## Abstract

Here we report the complete genome sequence of the bacterium *Paenibacillus polymyxa* CR1 (accession no. CP006941), which consists of one circular chromosome of 6,024,666 bp with 5,283 coding sequences (CDS), 87 tRNAs, and 12 rRNA operons. Data presented will allow for further insights into the mechanisms underpinning agriculturally and industrially relevant processes.

## GENOME ANNOUNCEMENT

*Paenibacillus polymyxa* is a species of Gram-positive, sporulating, facultative anaerobes that are widely distributed and have been isolated from various environmental samples (1–3). Recently, renewed interest has been shown in *P. polymyxa* as a potentially significant bacterium for its application in agriculture, industry, and medicine (4–10). So far, only three *P. polymyxa* genomes have been completely sequenced, *P. polymyxa* E681 (NC_014483), *P. polymyxa* SC2 (NC_014622), and *P. polymyxa* M1 (NC_017542) (11–13). Originally isolated from degrading corn tissues, *P. polymyxa* CR1 has demonstrated biocontrol, plant growth-promoting, solventogenic, and biomass-degrading properties (N. Nathoo, B. Weselowski, A. W. Eastman, and Z.-C. Yuan, unpublished data). Here we report the whole genome sequence in order to better elucidate the agriculturally and industrially relevant metabolic processes of *P. polymyxa* CR1.

Genomic DNA of *P. polymyxa* CR1 was isolated from culture growth for 24 h at 37°C in nutrient broth using a Sigma-Aldrich GenElute bacterial genomic DNA kit (product no. NA2120). Two libraries with an approximate insert size of 400 bp were prepared for sequencing using the NexteraXT DNA sample preparation kit for the small insert library and the Nextera mate pair sample preparation kit for the pair read library. The resulting sample libraries were purified and their quality evaluated by quantitative PCR (qPCR) and KAPA library quantification kit. These libraries were used as the templates for sequencing using a limited-cycle PCR using Nextera primers.

Sequencing was performed on the Illumina MiSeq platform, generating 2.9 million short insert reads and 140× coverage. Overlapping small insert library paired sequences and paired reads were merged, generating a 40× coverage read library which was assembled *de novo* using ABySS, Velvet, and SOAPdenovo (14–16). The best contig assembly from each program was assembled using CISA (17). This resulted in a final assembly of 38 contigs with sizes varying from 330 bp to 1.8 Mb. Where necessary, long and accurate PCR and Sanger sequencing with primer walking were performed to close remaining gaps between contigs.

The complete genome of *P. polymyxa* CR1 consists of one single circular chromosome of 6,024,666 bp, with a G+C content of 45.58%. The origin of replication was identified using Ori-Finder (18). Annotation was completed using the NCBI Prokaryote Genome Annotation Pipeline (19, 20). Genome annotation revealed 5,283 coding sequences, 87 tRNA loci, and 12 rRNA operons. Coding density is 82.2%, with an approximately equal distribution on both the forward and reverse strands (48.9% and 51.1% of genes, respectively).

Genes responsible for nitrogen fixation (*nif* genes) are present in the *P. polymyxa* CR1 genome, as well as genes responsible for plant hormone (indole-3-acetic acid) synthesis, biomass degradation, antimicrobial production, and butanol production (6, 21, 22). These genes corroborate our results, demonstrating biocontrol, plant growth promotion, biomass degradation, and biofuel production in *P. polymyxa* CR1 (Nathoo et al., unpublished). Further analysis and study are required to establish the intricacies of these complicated metabolic and signaling pathways (23).

In-depth analysis of the *P. polymyxa* CR1 genome will lay a solid foundation for understanding the synthetic pathways and underlying mechanisms. This knowledge will guide genetic modification of metabolic pathways to fully exploit the potential of *P. polymyxa* CR1 in agricultural and industrial applications.

### Nucleotide sequence accession number.

The sequence of *P. polymyxa* CR1 has been deposited in NCBI’s GenBank with the accession number CP006941.
